# Editorial: Brain Injury as a Neurodegenerative Disorder

**DOI:** 10.3389/fnhum.2015.00615

**Published:** 2016-01-05

**Authors:** Robin E. A. Green

**Affiliations:** ^1^Cognitive Neurorehabilitation Sciences Lab, Toronto Rehabilitation InstituteToronto, ON, Canada; ^2^Department of Psychiatry, Division of Neurosciences, University of TorontoToronto, ON, Canada

**Keywords:** neurodegeneration, traumatic brain injury, TBI, chronic TBI, atrophy, TBI as disease process, chronic traumatic encephalopathy, CTE: dementia in sports concussions

## Introduction

The acute stage of moderate-severe traumatic brain injury (TBI) entails the rapid unfolding of pathophysiological processes secondary to biomechanical damage that eventually stabilize, typically leaving a combination of focal damage (visible as encephalomalacia) and more widespread lesions, both to the white matter (known as traumatic axonal injury) and to the microvasculature of the brain (Povlishock and Katz, 2005). It has long been assumed that following resolution of these acute neuropathological events, that the brain then remains stable throughout the chronic stages of injury. However, a growing body of research, much of it from the groups represented in this special topic, has revealed ongoing losses to volume and white matter integrity of the brain (Bendlin et al., 2008; Ng et al., 2008; Farbota et al., 2012a,b; Adnan et al., 2013). Findings from these longitudinal studies do not appear to reflect simply the brain's *healing* for example, the process of gliosis or the resolution of edema. Rather, deterioration is observed in a number of studies between two time points that are well within the chronic stages of injury (e.g., Greenberg et al., 2008; Green et al., 2014), thereby representing progressive and possibly neurodegenerative changes.

With these important scientific developments in mind, the broad aims of this special topic of Frontiers in Human Neuroscience were three-fold: (i) To challenge the assumption of stability of the brain in chronic TBI and to advance a reconceptualization of moderate-severe TBI as a progressive, deteriorative disorder; (ii) to provide preliminary data on the characteristics and causes of deteriorative changes; and, (iii) to open a discussion about the clinical implications of these progressive changes observed in the chronic stages of TBI. The overarching goal of the issue is to stimulate further research into decline in the chronic stages of TBI, with a longer-term view to intervention research aimed at prevention or mitigation.

## The findings

The special topic focuses on research in patients with *moderate-severe TBI*, illustrating progressive losses to both white matter (Farbota et al., 2012a; Green et al., 2014) and gray matter (Green et al., 2014). A particularly concerning finding is the ubiquity of neurodegeneration: In one study (Green et al., 2014), the authors found significant atrophy in the chronic stages of injury in over 95% of their sample. A second line of related research in the issue focuses on the cumulative and chronic effects of *multiple milder* injuries (i.e., concussions/mild TBIs and sub-concussive blows), and the elevated risk of chronic traumatic encephalopathy (CTE) and other dementias (Hazrati et al., 2013; Tartaglia et al., 2014). Here, multiple mild TBIs are sustained—often in the context of professional contact sports, and in the second to fourth decades of life—but the neurodegeneration is typically observed many years after the last concussion has been sustained (though see McKee et al., 2013 for case studies of CTE in mid- and early-career athletes). Here again, the prevalence of neurodegeneration is noteworthy. In a recent study, 80% of the 85 brains of people with a history of high mild TBI exposure showed evidence of CTE (McKee et al., 2013). As noted by the authors, the study contained ascertainment biases, largely examining the brains of people with known neurological findings prior to death, for example. However, even if the findings represent an overestimate, they raise the specter of a considerably higher prevalence rate for neurodegeneration in this context than previously considered.

Discussing questions of prevalence, methodological challenges, and the history of CTE, Tartaglia et al. (2014) have provided a review of the CTE literature, one that is placed in the broader etiological context of tauopathies. On the same topic, Hazrati et al. (2013) have presented a post-mortem case series of retired professional football players, a population in whom a great deal of the CTE research has focused, with findings supporting the hypothesis that multiple concussions lead to neurodegeneration, but not exclusively to CTE.

In addition to these adult studies, there was preliminary evidence of neurodegeneration presented in the mini-review by Keightley et al. (2014). Interestingly, the totality of these findings (i.e., preliminary evidence for high incidence of neurodegeneration; and, neurodegeneration cutting across injury mechanisms [single severe vs. multiple mild] and across the age spectrum) suggests that neither a genetic nor demographic risk factor can fully account for neurodegeneration in TBI. Rather, the findings raise the question whether it is *post-injury* factors, set in motion by the injury (e.g., neuroinflammation—Johnson et al., 2013, or mood alterations), that may put many at risk, with “protective” factors potentially preventing or mitigating these effects in some. Bigler (2013b), who has studied the brain's instability after injury for over a decade (e.g., Tate and Bigler, 2000; Bigler, 2013a), examines in this special issue mechanisms of deterioration, and discusses the impact of TBI on age-typical brain development (mediated in part by the age at which the TBI is sustained) and on the aging process.

The challenges that lie ahead in understanding these mechanisms are illustrated well by an apparent paradox: Neurodegeneration in moderate-severe TBI is often observed within the first year or years of injury; thus, neural declines are often happening concurrently with behavioral *recovery* (see Bendlin et al., 2008). The co-occurrence of brain decline and behavioral recovery in moderate-severe TBI underscores that there are *multiple* mechanisms that the underlie brain changes during the chronic stages of injury, both beneficial and deleterious, and most likely interdependent.

With regard to the behavioral and clinical implications of this topic, Farbota et al. (2012a,b) have presented behavioral correlates of neurodegeneration (but also of recovery), while Miller et al. (2013) have revealed that environmental enrichment, and in particular cognitive enrichment, is negatively associated with volume loss in the hippocampi during the chronic stages of TBI—offering a new modifiable target of neuro rehabilitation (i.e., environmental enrichment for prevention of hippocampal atrophy in chronic TBI). Of note, Frasca et al. (2013) argue that the environments patients enter after clinical rehabilitation services have ended may contain reduced environmental enrichment, and thereby exacerbate neurodegeneration.

Lastly, in examining longitudinal degenerative change *in vivo*, it is essential to acknowledge the limitations in our imaging and analytic approaches. Junghoon et al. (2013) offer a potential solution to these challenges with a novel approach to MRI acquisition and analysis.

## Conclusions

We have presented a number of papers that illustrate the need to consider chronic moderate-severe TBI as a progressive, neurodegenerative disorder. This re-conceptualization opens new avenues for research, for example into the patterns and mechanisms of degeneration, and into protective factors and treatments. Clinically, the notion questions the prevailing approach to the delivery of clinical care, whereby services are concentrated in the early weeks and months of injury. If TBI patients are indeed declining in the chronic stages of injury, a re-evaluation of the current distribution of services is much needed.

## Funding

Canada Research Chairs Program 7006269; Canadian Institutes for Health Research (MOP 86704); National Sciences and Engineering Research (UT458054); Physicians Services Incorporated (12-43); ONF-REPAR (2007517).

### Conflict of interest statement

The author declares that the research was conducted in the absence of any commercial or financial relationships that could be construed as a potential conflict of interest.
